# Supplementary material for: mergem: merging, comparing, and translating genome-scale metabolic models using universal identifiers
Source: NAR Genom Bioinform. 2024 Feb 2;6(1):lqae010. doi: 10.1093/nargab/lqae010 (PMC10836943; doi:10.1093/nargab/lqae010)
Supplement: lqae010_Supplemental_Files [file lqae010_supplemental_files.zip › mergingGEMs_v4.0.manual.pdf]

mergem

Release 0.26.2

The Lobo Lab

Dec 15, 2023

CONTENTS

1 Installing the mergem package **3**

2 Using mergem to merge, compare, and translate models **5**
2.1 Command-line . . . 5
2.2 Python . . . 6
2.2.1 Merge models . . . 6
2.2.2 Other mergem functions . . . 7

3 Understanding mergem results **9**
3.1 Results from command-line execution . . . 9
3.1.1 Saving merged model . . . 9
3.1.2 Printing merging statistics . . . 9
3.2 Results from python package . . . 10

4 Visually comparing genome-scale metabolic models **11**

5 Updating Database ID mapping dictionaries **13**

6 Save ID mapping tables **15**
6.1 Saving mappers from the command-line . . . 15
6.2 Saving mappers using Python script . . . 15
6.2.1 Custom filenames for saving ID mappers . . . 15

7 Use examples **17**
7.1 Running on Command-line . . . 17
7.1.1 Merging reconstructions built using different templates . . . 17
7.2 Importing Python package . . . 18
7.2.1 Studying model versions . . . 18

8 Citing mergem **21**

9 Acknowledgements **23**

10 License **25**

11 Contact **27**

mergem is a Python library for merging, comparing, and translating genome-scale metabolic models. The library is publicly available via PyPI at https://pypi.org/project/mergem/ and can be pip installed. mergem can be used on the command-line and can also be imported within python scripts. The package can take models in various COBRApy compatible formats such as SBML, JSON, etc. and even COBRApy model objects, when the package is imported. The results of a single merge include the merged model, jaccard distances between all pairs of models, number of metabolites and reactions merged, and the mapping of each metabolite and reaction ID in the merged model to the corresponding metabolite or reaction IDs from each of the input models. Users can optionally select the objective, provide an output filename for the merged model, and translate the models to a different namespace.

For each input model, mergem converts the metabolite IDs into a common namespace using a database ID mapping dictionary. Reactions are compared using the participating metabolites (after conversion to common namespace). The metabolite ID mapping dictionary contains metabolite identifiers from various databases such as ModelSEED, KEGG, ChEBI, and MetaNetX that have been unified per metabolite. The dictionary thus allows for model metabolites to be compared more efficiently. The mapping dictionaries can be updated, during which the latest identifier information is downloaded from each database and identifiers representing the same metabolite are mapped to one another.

mergem is also available in the user-friendly application Fluxer, which produces tidy flux graphs that can visually compare the complete metabolic network from multiple models. Documentation for Fluxer based merging can be found on its tutorial page

CHAPTER

ONE

INSTALLING THE MERGEM PACKAGE

mergem can be installed from PyPI using pip installer:

```
pip install mergem
```

Upon installation, you can check the version using:

```
mergem --version
```

CHAPTER TWO

USING MERGEM TO MERGE, COMPARE, AND TRANSLATE MODELS

mergem can merge, compare, and translate genome-scale metabolic models. The command-line execution can take input models in various COBRApy compatible formats (SBML, JSON, YAML, and MAT). mergem can be imported into a python script and the merge function can take cobra objects in addition to filenames. A single objective function from any of the input models can be set as the objective for merged model. Alternatively, objective functions from all input models can be merged into a single function and set as the objective in the merged model. The metabolite and reaction IDS of the merged or standalone models can be translated to any of the database systems supported in mergem.

2.1 Command-line

Once mergem has been installed using pip, the following commands can be run on the command-line. The help argument displays all the options.

```
> mergem --help
Usage: mergem [INPUT_FILENAMES] [OPTIONS]

mergem takes genome-scale metabolic models as input, merges them into a
single model and saves merged model as .xml. Users can optionally select the
objective and provide an output filename for merged model.

Options:
-obj TEXT  Set objective: 'merge' all objectives (default) or 1, 2, 3...
         (objective from one of the input models)
-o TEXT    Save model as (filename with format .xml, .sbml, etc.)
-v         Print merging statistics
-up        Update ID mapping table
-s         Save ID mapping table as CSV
-e         Uses exact stoichiometry when merging reactions
-p         Consider protonation when merging reactions
-a         Extend annotations with mergem database of metabolites and reactions
-t         Translate metabolite and reaction IDs to a target namespace (chebi, metacyc,␣
↪kegg, reactome, metanetx, hmdb, biocyc, bigg, seed, sabiork, or rhea)
--version  Show the version and exit.
--help     Show this message and exit.
```

For merging two models and setting objective of merged model from first model, use:

```
mergem model1.xml model2.xml
```

The `-obj` argument can be used to set the objective function of merged model. Allowed values include `merge` to merge input model objective functions (default) or an integer representing the objective function from the model in order of

input (1, 2, 3, ..):

```
mergem model1.xml model2.xml -obj 1
```

To print merge statistics, append the -v argument:

```
mergem model1.xml model2.xml -v
```

Output model filename can be provided using the -o argument followed by desired output filename with file format specified in the extension (.xml, . . .):

```
mergem model.xml model2.xml -o mergedmodel.xml
```

Save the ID mapping table as a CSV file by using the -s argument:

```
mergem model1.xml model2.xml -s
```

By default, reactions are merged when they have both a similar set of reactants and a similar set of products, without comparing their stoichiometry. To merge reactions only when they have the same exact stoichiometry in their reactants and products, use the -e argument:

```
mergem model1.xml model2.xml -e
```

By default, reactions are compared ignoring the hydrogen and proton metabolites. To consider also the hydrogen and proton metabolites when comparing reactions, use the -p argument:

```
mergem model1.xml model2.xml -p
```

Metabolite and reaction annotations are merged from all input models. In addition, mergem can extend these annotations using the mergem database. For extending the annotations using mergem dabase, use the -a argument:

```
mergem model1.xml model2.xml -a
```

Mergem can translate the metabolite and reaction IDs to another database system when using the -t argument:

```
mergem model1.xml -t chebi
```

2.2 Python

2.2.1 Merge models

Import the mergem package to use its modules within a python script:

```
import mergem
```

Provide the list of models to be merged:

```
results = mergem.merge(input_models, set_objective='merge', exact_sto=False, use_
↪prot=False, extend_annot=False, trans_to_db=None)
merged_model = results['merged_model']
jacc_matrix = results['jacc_matrix']
num_met_merged = results['num_met_merged']
num_reac_merged = results['num_reac_merged']
```

(continues on next page)

(continued from previous page)

```
met_sources = results['met_sources']
reac_sources = results['reac_sources']
```

- `input_models` is a list of one or more COBRApy model objects or strings specifying file names.
- `set_objective` specifies if the objective functions are merged ('merge') or copied from a single model (specifying the index of the model: '1', 2', '3', etc.).
- `exact_sto` use exact stoichiometry when merging reactions.
- `use_prot` consider hydrogen and proton metabolites when merging reactions.
- `add_annot` add additional metabolite and reaction annotations from mergem dictionaries.
- `trans_to_db` translate metabolite and reaction IDs to a target database (chebi, metacyc, kegg, reactome, metanetx, hmdb, biocyc, bigg, seed, sabiork, or rhea)
- `results` a dictionary with all the results, including:
- `merged_model` the merged model.
- `jacc_matrix` metabolite and reaction jaccard distances.
- `num_met_merged` number of metabolites merged.
- `num_reac_merged` number of reactions merged.
- `met_sources` dictionary mapping each metabolite ID in the merged model to the corresponding metabolite IDs from each of the input models.
- `reac_sources` dictionary mapping each reaction ID in the merged model to the corresponding reaction IDs from each of the input models.

2.2.2 Other mergem functions

The following functions can also be imported from mergem:

```
from mergem import translate, load_model, save_model, map_localization, map_metabolite_
↪univ_id, map_reaction_univ_id,
                    get_metabolite_properties, get_reaction_properties, update_id_mapper
```

`translate(input_model, trans_to_db)` translates a model to another target database.

`load_model(filename)` loads a model from the given filename/path.

`save_model(cobra_model, file_name)` takes a cobra model as input and exports it as file file_name.

`map_localization(id_or_model_localization)` converts localization suffixes into common notation.

`map_metabolite_univ_id(met_id)` maps metabolite id to metabolite universal id.

`map_reaction_univ_id(reac_id)` maps reaction id to metabolite universal id.

`get_metabolite_properties(met_univ_id)` retrieves the properties of a metabolite using its universal id

`get_reaction_properties(reac_univ_id)` retrieves the properties of a reaction using its universal id

`update_id_mapper(delete_database_files)` updates and build mergem database. It will download the latest source database files, merge the identifiers based on common properties, and save the mapping mapping tables and information internally. This process can take several hours. The parameter specifies if the downloaded intermediate database files are deleted after the update (saves disk space but the next update will take longer; dafault is True).

All the functions can be imported at once with:

```
from mergem import *
```

CHAPTER
THREE

UNDERSTANDING MERGEM RESULTS

3.1 Results from command-line execution

3.1.1 Saving merged model

When no output filename and format is given, the command-line execution of mergem without any arguments produces a .xml file containing the merged model in SBML format with filename that's a concatenation of the SBML model IDs of input model files separated by an underscore "_". mergem uses COBRApy to save the merged model and thus can save models in the SBML, JSON, and MATLAB formats.

To change the output filename and format, provide the `-o` argument to the mergem command:

```
mergem model1.xml model2.xml -o myfilename.xml
```

In the above example, mergem will save the merged model as `myfilename.xml` in current working directory.

3.1.2 Printing merging statistics

To print statistics on the input models and the merging, enter the `-v` argument:

```
mergem model1.xml model2.xml -v
```

Once the models are merged, input model Jaccard distances and the number of metabolites and reactions merged are printed to the console.

Jaccard distances are printed as a matrix with the metabolic and reaction Jaccard distances of pairs of input models. The matrix follows the format shown below:

$$\mathrm{JM}(G_1, \ldots, G_n) = \begin{bmatrix} 0 & \mathrm{J}(M_1, M_2) & \ldots & \mathrm{J}(M_1, M_n) \\ \mathrm{J}(R_2, R_1) & 0 & \ddots & \vdots \\ \vdots & \ddots & 0 & \mathrm{J}(M_n, M_n) \\ \mathrm{J}(R_n, R_1) & \ldots & \mathrm{J}(R_n, R_n) & 0 \end{bmatrix}$$

where $J(M_i, M_j)$ and $J(R_i, R_j)$ represent the metabolite and reaction Jaccard distances between models i and j.

Statistics are printed in the format as shown in the example below:

```
Jaccard distance matrix: [[0, 0], [0, 0]]
Mets merged: 72
Reacs merged: 94
```

In the above case, two models were merged. A Jaccard distance of 0 indicates close match with reference model. Thus the two models are an exact match of each other. The result also shows that 72 metabolites and 94 reactions were merged between the two models.

3.2 Results from python package

Merging models using the mergem package on a python script returns a dictionary of results including the merged model, Jaccard distances, number of merged metabolites and reactions, and a dictionary containing the indices of input models within which each metabolite and reaction were found.

```
from mergem import merge

merge_results = merge([model1, model2])
```

Individual results can be accessed using the dictionary keys `merged_model` , `jacc_matrix` , `num_met_merged` , `num_reac_merged` , `met_sources` , and `reac_sources` as shown below:

```
merged_model_obj = merge_results['merged_model']
jaccard_distances = merge_results['jacc_matrix']
num_mets_merged = merge_results['num_met_merged']
num_reacs_merged = merge_results['num_reac_merged']
metabolite_sources = merge_results['met_sources']
reaction_sources = merge_results['reac_sources']
```

CHAPTER

FOUR

VISUALLY COMPARING GENOME-SCALE METABOLIC MODELS

Visualizing the components of each input model with different colors can help compare and visually identify the components that are common between all models or unique to a single model. The large size of genome-scale metabolic models makes it more challenging to visualize such relationships.

Fluxer is a user-friendly web-application that can visualize genome-scale metabolic models using various graphs and layouts. We have made mergem available on Fluxer so that users can merge models while also visualizing their components. The Fluxer interface can be used to interact with the merged model, simulate knockouts in the merged model and even download the final metabolic model (in SBML and graphML formats) and its graphs (in PNG and SVG formats).

The Fluxer tutorial page provides details on how to merge models on Fluxer, how to interpret the results and customize the networks generated.

CHAPTER

FIVE

UPDATING DATABASE ID MAPPING DICTIONARIES

The mergem algorithm uses database ID mapping dictionaries to map model metabolite identifiers into an internal identifier that allows for comparison and merging of metabolites. Updating the mapping dictionary involves downloading the latest identifier information from databases such as SEED, MetaNetX, KEGG, etc and linking identifiers across databases if they represent the same metabolite, after checking for matching properties. The update process can take a few hours and depends on the internet connection.

The `-up` argument can be used to update the ID mapping pickles on the command line:

```
mergem -up
```

When importing mergem into a python script, the `update_id_mapper()` function can be called to update the mappping dictionaries as shown below:

```
import mergem

mergem.update_id_mapper()
```

CHAPTER

SIX

SAVE ID MAPPING TABLES

6.1 Saving mappers from the command-line

The database ID mapping tables can be downloaded as csv files. Each file contains all the universal IDs for either metabolites or reactions followed by the corresponding cross-referenced identifiers in other databases.

The ID mapping tables are saved as `mergem_univ_id_mapper_metabolites.csv` and `mergem_univ_id_mapper_reactions.csv` in the working directory.

The `-s` argument can be used to save the ID mapping tables on the command line:

```
mergem -s
```

6.2 Saving mappers using Python script

When importing mergem into a python script, the `save_mapping_tables()` function can be called to save the mapping tables as shown below:

```
import mergem

mergem.save_mapping_tables()
```

6.2.1 Custom filenames for saving ID mappers

The mapping table filenames can be customized by providing the desired filenames as input to the function.

```
mergem.save_mapping_tables('custom_met_mapper_filename.csv', 'custom_reac_mapper_
↪filename.csv')
```

CHAPTER

SEVEN

USE EXAMPLES

7.1 Running on Command-line

7.1.1 Merging reconstructions built using different templates

To compare the effect of reconstruction parameters, we merged two P. putida draft reconstructions built on ModelSEED using either a gram-positive or core template.

```
mergem -v MS1_PPU.sbml MS2_PPU.sml -o MS1_MS2_merged_PPU.xml
```

Results from merging the two reconstructions on the command-line using mergem are shown below.

```
Merging models complete. Merged model saved as MS1_MS2_merged_PPU.xml
Jaccard distance matrix: [[0, 0.02425267907501405], [0.049657534246575374, 0]]
Metabolites merged: 1730
Reactions merged: 1666
```

The figure below shows the same merging on the web-application Fluxer and can be accessed here.

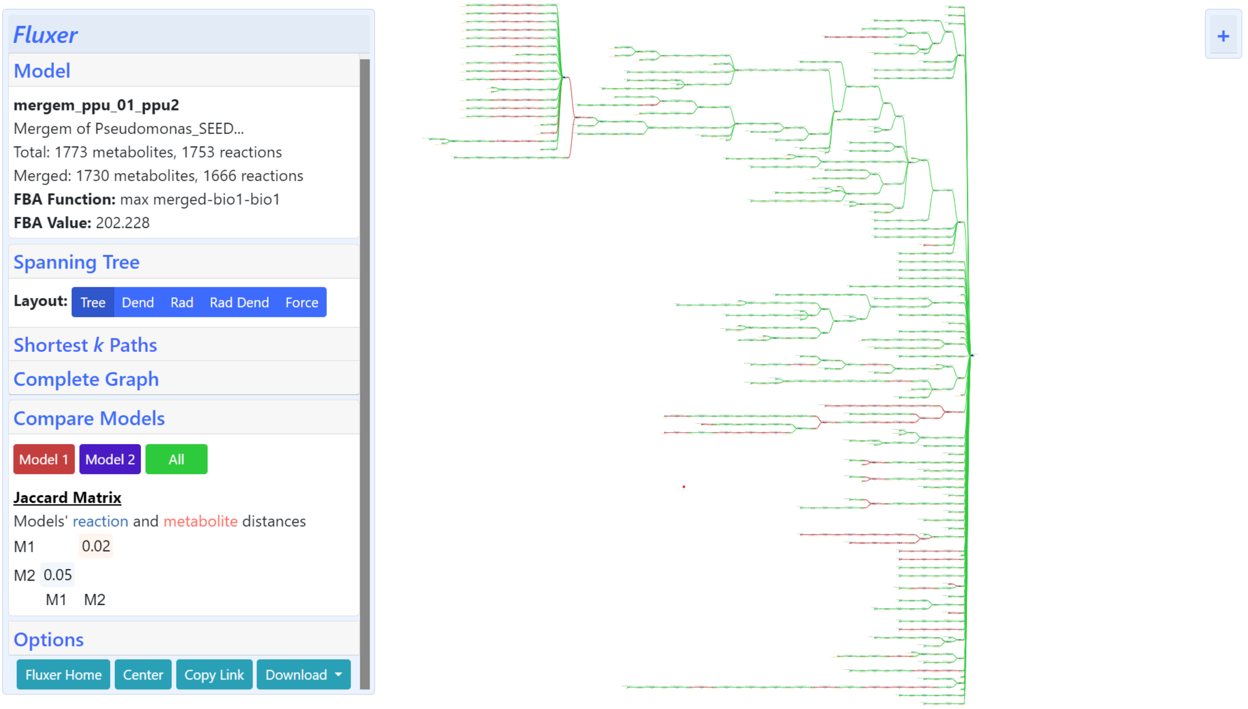

7.2 Importing Python package

7.2.1 Studying model versions

Different versions of models can be compared to analyze elements that were added or removed during update. The results of comparing three versions of a P. putida KT2400 model using mergem on the Python console are shown below:

```
import mergem

input_models_list = ['models/iJN1463.xml', 'models/iJN746.xml', 'models/MNX_iJN746.sbml']
merge_results = mergem.merge(input_models_list, set_objective='merge')

print('Number of metabolites in merged model: ', len(merge_results['merged_model'].
↪metabolites))
print('Number of reactions in merged model: ', len(merge_results['merged_model'].
↪reactions))

print("Jaccard matrix: \n", merge_results['jacc_matrix'])

print('Number of metabolites merged between input models: ', merge_results['num_met_
↪merged'])
print('Number of reactions merged between input models: ', merge_results['num_reac_merged
↪'])
```

Running the above script produces the following output:

```
Number of metabolites in merged model:  2186
Number of reactions in merged model:  3001
Jaccard matrix:
[[0, 0.5976168652612283, 0.5986270022883295], [0.6765588529509836, 0, 0.
↪006586169045005531], [0.679, 0.01526717557251911, 0]]
Number of metabolites merged between input models:  1783
Number of reactions merged between input models:  2024
```

The same result including the Jaccard matrix can be visualized on Fluxer as shown below:

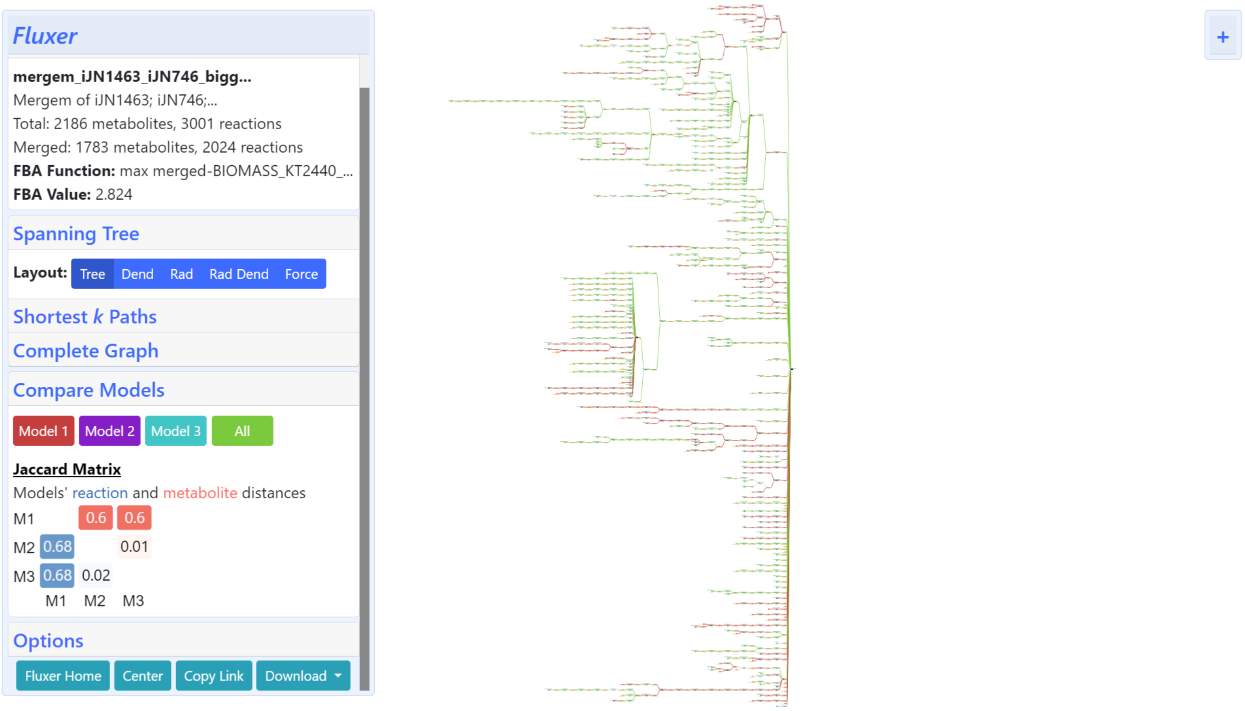

The above Fluxer result can be accessed here.

CHAPTER

EIGHT

CITING MERGEM

Please cite mergem using:

mergem: merging and comparing genome-scale metabolic models using universal identifiers
A. Hari, D. Lobo.
bioRxiv , doi:10.1101/2022.07.14.499633 , 2022

Contributors:

Archana Hari
Arveen Zarrabi
Daniel Lobo

CHAPTER

NINE

ACKNOWLEDGEMENTS

We thank The Lobo Lab for their help with software testing and The University of Maryland Baltimore County for its resources and support.

We also thank the following packages and databases for their public resources:

1. COBRApy
2. Click
3. ModelSEED
4. KBase
5. MetaNetX
6. KEGG
7. ChEBI

CHAPTER

TEN

LICENSE

This package is under GNU GENERAL PUBLIC LICENSE. The package is free for use without any express or implied warranty. In no event will the authors be held liable for any damages arising from the use of this software. Permission is granted to anyone to use this software for any purpose, subject to the following restrictions:

1. The origin of this software and database must not be misrepresented; you must not claim that you wrote the original software.
2. If you use this software and/or database in a work (any production in the scientific, literary, and artistic domain), an acknowledgment and citation (see publication above) in the work is required.
3. This notice may not be removed or altered from any distribution.

CHAPTER

ELEVEN

CONTACT

Please contact The Lobo Lab with any questions and suggestions.
