# Supplementary material for: mergem: merging, comparing, and translating genome-scale metabolic models using universal identifiers
Source: NAR Genom Bioinform. 2024 Feb 2;6(1):lqae010. doi: 10.1093/nargab/lqae010 (PMC10836943; doi:10.1093/nargab/lqae010)
Supplement: lqae010_Supplemental_Files [file lqae010_supplemental_files.zip › mergingGEMs_v4.0_SupplementaryTables.pdf]

mergem: merging, comparing, and translating genome-scale metabolic models using universal identifiers

Archana Hari, Arveen Zarrabi, and Daniel Lobo

Supplementary Tables

Contents

Supplementary Table 1	Number of metabolite and reaction IDs cross-referenced by the universal mapping system.
Supplementary Table 2	Number and percentage of metabolites and reactions merged between pairs of reconstructions using different tools.
Supplementary Table 3	Potential metabolic gaps in ModelSEED reconstruction for *L. plantarum* and candidates for filling the gaps.
Supplementary Table 4	Fluxer URLs for each of the models presented in the main figures.

Supplementary Table 1. Number of metabolite and reaction IDs cross-referenced by the universal mapping system. Rows and columns indicate source and target databases, respectively. Diagonal indicates total number of IDs in each database.

Metabolites	MetaNetX	ModelSEED	KEGG	BiGG	Reactome	ChEBI	BioCyc	HMDB	MetaCyc	SabioRK	LIPID MAPS	SLM
**MetaNetX**	1301326	45828	48657	13545	5015	143094	4460	117719	31509	3777	47472	779015
**ModelSEED**	33994	33995	19403	3915	1445	21604	2222	5807	18284	1436	3391	1082
**KEGG**	41804	20716	42094	2803	1482	23030	1956	6222	8949	1383	2680	647
**BiGG**	9031	3623	2490	9034	1043	3145	1945	2235	2448	649	1038	615
**Reactome**	5460	2413	2281	2050	5460	2942	1609	1928	2085	744	789	646
**ChEBI**	154084	34595	33915	11931	6242	176752	9577	25918	23885	4622	12696	8914
**BioCyc**	1879	1878	1640	1858	727	1786	1879	1244	1742	506	448	240
**HMDB**	116169	7075	6477	3827	1816	19149	2601	116169	5345	1295	8046	24282
**MetaCyc**	24146	17934	8345	2518	1190	12016	1895	4019	24146	1255	2470	974
**SabioRK**	2532	1265	1113	613	354	1340	480	786	1131	2532	295	125
**LIPID MAPS**	45473	3385	2601	1075	543	9208	516	7646	2588	331	45473	11976
**SLM**	777956	1022	584	559	332	6683	246	23973	982	126	11867	777956

Reactions	MetaNetX	ModelSEED	KEGG	BiGG	MetaCyc	SabioRK	Rhea
**MetaNetX**	77403	38278	12683	24451	19628	9790	13453
**ModelSEED**	43850	44020	11070	9173	16297	4597	8235
**KEGG**	11330	7595	11330	1976	4996	1711	4062
**BiGG**	87732	31338	12554	90677	15615	11599	13782
**MetaCyc**	18546	11661	4979	2180	18546	1727	4788
**SabioRK**	8953	2260	1752	1456	1758	8953	1603
**Rhea**	48146	19905	15704	7189	18577	6033	48146

Note: For this analysis, all files were downloaded on April 17, 2023 and the version of each database, when provided, were as follows:

MetaNetX	ModelSEED	KEGG	BiGG	Reactome	ChEBI	BioCyc	HMDB	MetaCyc	SabioRK	LIPID MAPS
4.4	2.6.1	98	1.6.0	77	203	25.0.0	4.0.0	25.0.0	5/28/2021	5/28/2021

Supplementary Table 2. Number and percentage of metabolites and reactions merged between pairs of models using different tools. "-" indicates tool failure when loading or merging the models. AU: AuReMe, CA: CarveMe, MS: ModelSEED, MD: MetaDraft, PT: Pathway Tools, and RA: RAVEN

**Metabolites (number)**	**COBRApy**	**MetaNetX**	**iMET**	***mergem***
AU + CA	714	506	-	714
AU + MD	785	-	-	785
AU + MS	0	-	365	536
AU + PT	1	518	44	499
AU + RA	0	-	-	565
CA + MD	698	-	701	698
CA + MS	0	-	-	801
CA + PT	1	392	-	638
CA + RA	0	-	19	634
MD + MS	0	-	-	529
MD + PT	1	-	-	491
MD + RA	0	-	17	550
MS + PT	0	-	0	723
MS + RA	0	-	-	801
PT + RA	0	-	-	844

**Metabolites (%)**	**COBRApy**	**MetaNetX**	**iMET**	***mergem***
AU + CA	60.6	42.9		60.6
AU + MD	98.2			98.2
AU + MS	0.0		28.2	41.4
AU + PT	0.1	30.0	2.5	28.9
AU + RA	0.0			47.6
CA + MD	59.9		60.2	59.9
CA + MS	0.0			48.3
CA + PT	0.0	18.7		30.5
CA + RA	0.0		1.2	40.8
MD + MS	0.0			41.4
MD + PT	0.1			28.6
MD + RA	0.0		1.4	46.9
MS + PT	0.0		0.0	32.7
MS + RA	0.0			48.1
PT + RA	0.0			40.1

**Reactions (number)**	**COBRApy**	**MetaNetX**	**iMET**	***mergem***
AU + CA	700	752	-	784
AU + MD	771	-	-	772
AU + MS	0	-	629	569
AU + PT	0	319	20	625
AU + RA	3	-	-	469
CA + MD	704	-	714	713
CA + MS	0	-	-	901
CA + PT	0	678	-	781
CA + RA	0	-	142	574
MD + MS	0	-	-	571
MD + PT	0	-	-	626
MD + RA	3	-	125	470
MS + PT	0	-	128	771
MS + RA	0	-	-	626
PT + RA	0	-	-	675

**Reactions (%)**	**COBRApy**	**MetaNetX**	**iMET**	***mergem***
AU + CA	45.7	49.1		51.2
AU + MD	99.5			99.7
AU + MS	0.0		49.8	45.1
AU + PT	0.0	17.9	1.1	35.1
AU + RA	0.3			42.1
CA + MD	45.9		46.5	46.5
CA + MS	0.0			44.6
CA + PT	0.0	26.7		30.7
CA + RA	0.0		7.6	30.6
MD + MS	0.0			45.1
MD + PT	0.0			35.0
MD + RA	0.3		11.2	42.1
MS + PT	0.0		5.6	33.9
MS + RA	0.0			39.0
PT + RA	0.0			31.8

Note: Percentages were calculated as $100 * \frac{2*N_m}{N_1+N_2}$, where N_M represents the number of metabolites or reactions merged and N_1 and N_2 indicates the total number of metabolites or reactions in the first or second model, respectively.

Supplementary Table 3. Nine potential gaps and fillers identified in ModelSEED reconstruction for *L. plantarum*. Manually curated model for *L. reuteri* from (Kristjansdottir *et al.*, 2019). ModelSEED reconstruction and manually curated *L. plantarum* models from (Mendoza *et al.*, 2019).

**Reaction ID**		**Reaction name**
L. reuteri	***L. plantarum***	
ABTA	ABTA	4-Aminobutyrate transaminase
GLUT6	GLUt2r	L-Glutamate transport via proton symport
PUNP1	PUNP1	Purine nucleoside phosphorylase-Adenosine
PUNP3	PUNP3	Purine nucleoside phosphorylase-Guanosine
PUNP5	PUNP5	Purine nucleoside phosphorylase-Inosine
ALATA_Lr	ALATA_Lr	Alanine transaminase
BTNt2i	BTNt2i	Biotin uptake
MALTt	MALTt2	Maltose transport via proton symport
HYPOE	HYPOE	Pyridoxamine-5'-phosphate phosphohydrolase

Supplementary Table 4. URLs for the models presented in each of the main figures, which can be used to access, analyze, and download the models from Fluxer web application.

Figure	Fluxer URL
3	https://fluxer.umbc.edu/model?id=1fb050032ca2e1dce96190af995cadb92b40f184_3963a7426d7d662b0f404284c397e13227361d88_0335833002b8337f8440cf1f09a41c9aa6d3268f_obj_merge
5	https://fluxer.umbc.edu/model?id=0335833002b8337f8440cf1f09a41c9aa6d3268f_3963a7426d7d662b0f404284c397e13227361d88_obj_merge
6	https://fluxer.umbc.edu/model?id=7fe8a8e65427f5f30412cc3341b5ec596e956f42_8483528fcd5891b944d53a6e4f61214acb596f42_obj_merge
7	https://fluxer.umbc.edu/model?id=631396a6150696a9af6f26b9bca4c9b63343b99f_f25b134379c57bba2fe256c7ad737f8729cb2864_obj_merge
8A	https://fluxer.umbc.edu/model?id=fb168cce92cd64a84ecbbf4e13dedad56adadedb_bd1605b71563d7dde27f5f57552651d9934c0333_obj_1
8B	https://fluxer.umbc.edu/model?id=6002faacf35a41f4f1b4132f045dd6ff7071d31e_bd1605b71563d7dde27f5f57552651d9934c0333_obj_1
9	https://fluxer.umbc.edu/model?id=f4f30bd4265c5734d98b719ce39e0dbbd5d4ecfb_0bab56f8be08a3d62f24dd16c00b6b01fd85cb27_7fe25baa10e0f3ee212b5b8b4edc4742e5ebfd8b_obj_merge
10	https://fluxer.umbc.edu/model?id=5f84c87ff0449775359f3adb1ddfc75d6e96202d_f4f30bd4265c5734d98b719ce39e0dbbd5d4ecfb_0bab56f8be08a3d62f24dd16c00b6b01fd85cb27_obj_merge
11	https://fluxer.umbc.edu/model?id=e49e478a9849adcb1cb7e03409de63e470b2880d_a84d145f63822725400bac076aa22078d6a77189_5f84c87ff0449775359f3adb1ddfc75d6e96202d_obj_1
